# Validation of a high performance functional assay for individual radiosensitivity in pediatric oncology: a prospective cohort study (ARPEGE)

**DOI:** 10.1186/s12885-018-4652-7

**Published:** 2018-07-06

**Authors:** Valérie Bernier-chastagner, Liza Hettal, Véronique Gillon, Laurinda Fernandes, Cécile Huin-schohn, Marion Vazel, Priscillia Tosti, Julia Salleron, Aurélie François, Elise Cérimèle, Sandrine Perreira, Didier Peiffert, Pascal Chastagner, Guillaume Vogin

**Affiliations:** 1 Department of radiation therapy, Institut de Cancérologie de Lorraine, Vandoeuvre Les Nancy, France; 2 UMR 7365 CNRS-UL, IMoPA, Vandoeuvre Les Nancy, France; 3 Clinical Trials Promotion Unit, Institut de Cancérologie de Lorraine, Vandoeuvre-Les-Nancy, France; 4 Biostatistics Unit, Institut de Cancérologie de Lorraine, Vandoeuvre Les Nancy, France; 5 Basic Research Laboratory, Institut de Cancérologie de Lorraine, Vandoeuvre Les Nancy, France; 6 Neolys Diagnostics R&D department, Lyon, France; 7 Department of Pediatric Oncology, CHRU Nancy, Vandoeuvre Les Nancy, France

**Keywords:** Pediatric oncology, Radiotherapy, Radiosensitivity, Toxicity, Biomarker, Predictive assay

## Abstract

**Background:**

Approximately 900 children/adolescents are treated with radiotherapy (RT) every year in France. However, among the 80% of survivors, the cumulative incidence of long-term morbidity – including second malignancies - reach 73.4% thirty years after the cancer diagnosis. Identifying a priori the subjects at risk for RT sequelae is a major challenge of paediatric oncology. Individual radiosensitivity (IRS) of children/adolescents is unknown at this time, probably with large variability depending on the age when considering the changes in metabolic functions throughout growth. We previously retrospectively showed that unrepaired DNA double strand breaks (DSB) as well a delay in the nucleoshuttling of the pATM protein were common features to patients with RT toxicity. We aim to validate a high performance functional assay for IRS prospectively.

**Methods/design:**

ARPEGE is a prospective open-label, non-randomized multicentre cohort study. We will prospectively recruit 222 children/adolescents who require RT as part of their routine care and follow them during 15 years. Prior RT we will collect blood and skin samples to raise a primary dermal fibroblast line to carry out in blind the IRS assay. As a primary objective, we will determine its discriminating ability to predict the occurrence of unusual early skin, mucous or hematological toxicity. The primary endpoint is the measurement of residual double-strand breaks 24 h after ex vivo radiation assessed with indirect immunofluorescence (γH2AX marker). Secondary endpoints include the determination of pATM foci at 10 min and 1 h (pATM marker) and micronuclei at 24 h. In parallel toxicity including second malignancies will be reported according to NCI-CTCAE v4.0 reference scale three months of the completion of RT then periodically during 15 years. Confusion factors such as irradiated volume, skin phototype, previous chemotherapy regimen, smoking, comorbities (diabetes, immunodeficiency, chronic inflammatory disease...) will be reported.

**Discussion:**

ARPEGE would be the first study to document the distribution of IRS in the pediatric subpopulation. Screening hypersensitive patients would be a major step forward in the management of cancers, opening a way to personalized pediatric oncology.

**Trial registration:**

ID-RCB number: 2015-A00975–44, ClinicalTrials.gov Identifier: NCT02827552 Registered 7/6/2016.

## Background

2400 children and adolescents are newly diagnosed with cancer every year in France with an average age at diagnosis of 5 years [1, 2]. Although most protocols are currently attempting to limit the use of radiotherapy (RT) in this population, ionizing radiations remain a keystone in the management of approximately 900 patients who suffer from brain tumors, Hodgkin’s lymphoma, leukemia, soft tissues sarcomas, neuroblastomas, nephroblastomas or retinoblastomas [3].

However, among the 80% of survivors, the cumulative incidence of long-term morbidity – including second malignancies - reach 73.4% thirty years after the cancer diagnosis, with a cumulative incidence of 42.4% for severe, disabling, or life-threatening toxicities or specific death [4]. A major socio-economic impact is noticed (schooling issues, parental mobilization, unemployment, hospitalization, expensive symptomatic treatment, impoverishment, etc.). As 5–10% of adults treated with RT experience overreactions [5, 6], the distribution of individual radiosensitivity (IRS) in the pediatric population has never been described, with probably large age-related variability when considering all changes in metabolic functions throughout growth and tumor predispositions involving DNA repair pathways [7]. Only short series have been reported [8, 9]. Identifying those patients prior treatment would therefore have sound positive clinical implications such as the perspective of personalized therapy.

Predictive assays of RT toxicity are subject to a lack of universality, reproducibility and specificity, and are time consuming [10] so that there is no gold standard. An Increasing number of studies show that double-strand breaks (DSB) are the DNA damage most closely correlated with cell lethality and *in fine* toxicity if not repaired on the one hand, or genomic instability and cancer risk if misrepaired on the other hand [11, 12].

From 2003, the INSERM UMR1052 radiobiology group has elaborated a collection of primary skin fibroblast lines from patients (the majority of adults) with DNA repair deficiencies or who experienced RT toxicity on various tissues, with various severity and different post RT free intervals. The RT-induced distribution of candidate DSB recognition and repair proteins was measured with an indirect immunofluorescence (IIF) assay [13, 14] leading to a general classification of Human IRS based on the rate of unrepaired DSB 24 h after ex vivo irradiation (γH2AX marker). This last study showed further that a delay in the nucleoshuttling of the pATM protein, which is involved in the recognition of the DSB, was a common feature to patients with overreaction (OR) [15]. The maximal number of pATM foci between 10 min and 1 h post irradiation (pATM_max_) was found to be the parameter with the best correlation with each OR severity grade, independently of tumor localization and of the early or late nature of reactions. When taken as a binary predictive assay with the optimal cut-off value of 35 pATM foci, pATM_max_ foci showed promising predictive performances on a retrospective study, with an AUC of 0.97, a PPV of 99%, a specificity of 92% and a sensitivity of 100% [16].

We designed a prospective open-label, non-randomized multicenter cohort study to address the distribution of IRS and validate the performance of the IRS assay in the pediatric subpopulation. We will prospectively recruit 222 children/adolescents who require RT as part of their care path and follow them during 15 years to describe the specific morbidity including second malignancies. Prior RT we will collect blood and skin samples to raise a primary dermal fibroblast line to carry out the IRS assay – the result of which will have no impact on the RT prescription.

## Methods/design

### Aim, design and setting

#### Objectives

ARPEGE aims to explore the distribution of IRS in the pediatric population and to determine prospectively the discriminating ability of an IRS assay to predict the occurrence of early cutaneous, mucosal or hematological RT toxicity qualified as unusual in children/adolescents treated with RT for cancer.

The short term secondary objectives consist in: 1) identifying thresholds for each biomarker to predict the occurrence of unusual early toxicity in order to define IRS groups, 2) comparing the discriminating ability of IIF biomarkers (pATM and γH2AX on the one hand and micronuclei on the other hand), 3) developing a multivariate predictive model combining biomarkers.

The long-term secondary objectives consist in: 1) identifying the discriminating ability of biomarkers to predict the occurrence of Grade 3–4 late toxicities, and thresholds for each biomarker, 2) describing IRS biomarkers in the subset of patients developing secondary malignancies, 3) investigating the correlation between the severity of early toxicity and the occurrence of late toxicity (including secondary malignancies).

#### Endpoints

The primary endpoint is the skin fibroblast radiosensitivity defined as the residual DSB 24 h after ex vivo radiation assessed with IIF (γH2AX marker). Unusual early cutaneous, mucosal or hematological toxicity occurs within 3 months after RT and is defined by any of the following features appreciated with CTCAE v4.0 morbidity scale:

- Grade 2 or higher occurring at low doses (first week of treatment) or
- Grade 3–4 for more than 4 weeks after completion of RT and / or requiring surgery [17].

The discriminating ability of this biomarker to predict the occurrence of early toxicity will be assessed by the area under the receiver operating characteristic (ROC) curve.

Kinetic data on other DSB repair proteins are collected in order to refine the classification of IRS as secondary endpoints. The other biomarkers studied are therefore: 1) the number of pATM foci 10 min and 1 h post irradiation, 2) the average number of micronuclei per cell 24 h after irradiation (control IRS assay).

Late toxicity including second malignancies occurs at least 3 months after the completion of RT. Severe sequelae are defined by any grade 3–4 adverse effect with a progression lasting more than 90 days [17].

### Participants and recruitment

#### Inclusion/non-inclusion criteria

All children and adolescents treated with a curative intent in pediatric oncology and radiotherapy departments of the Grand-Est and Burgundy-Franche-Comté regions, France participating in the GE-HOPE network (i.e. Nancy, Reims, Dijon, Strasbourg and Besançon) as well as Lyon and Toulouse may be included according to the following inclusion criteria: 1) age < 18 years, 2) indication of RT as part of the local control strategy on the primary tumor, 3) standard fractionation (1.8–2.2 Gy/fraction, 5 sessions/week) irrespective of the technique and particle used, 4) patient affiliated to social security insurance, 5) patient and / or holder(s) of parental authority signed a written informed consent.

Exclusion criteria are: 1) contra-indication to skin biopsy, 2) contra-indication to RT, 3) RT in a palliative intent, 4) Previous irradiation in the same anatomic site (re-irradiation), 5) hypofractionation, 6) Impossible follow-up, 7) Persons deprived of liberty or under supervision.

### Recruitment

**Fig. 1:**
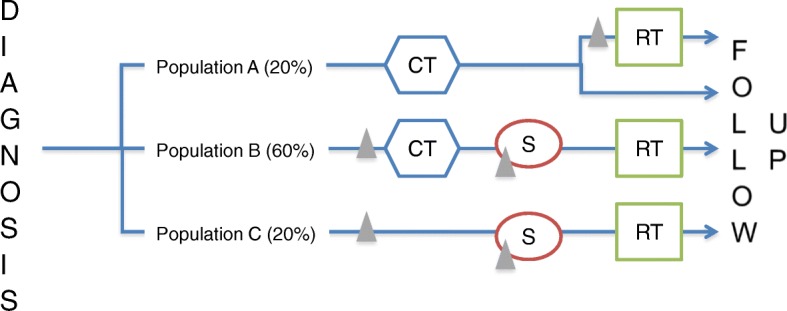
Recruitment modalities and timing of the biopsy. CT: chemotherapy, RT: radiotherapy, Δ: biopsy. • Population A: definitive RT only if the response to neoadjuvant CT in unsatisfactory. • Population B: neoadjuvant CT before RT, eventually followed with CT. • Population C: definitive RT not preceded by CT

The participation to ARPEGE will be proposed as soon as the indication of RT is collegially validated in the care path of the patient. Three modalities of inclusion are permitted independently of surgery when required (Fig. 1):

- about 20% of patients could benefit from RT only if the response to neoadjuvant CT in unsatisfactory. This data is known late. The biopsy would therefore be sampled just before the RT (modality A, mainly lymphomas)
- 60% of patients could benefit from neoadjuvant CT before RT (modality B, sarcomas, neuroblastomas, nephroblastomas, medulloblastomas…)
- 20% of patients could benefit from definitive RT not preceded by CT (modality C, mainly brain tumors)

In the latter two cases, the biopsy will be anticipated.

### Data collection methods

All patients providing written informed consent to participate in the study are asked to complete a medical history. Clinical data that will be obtained in the ARPEGE study include patient-related data (age, height, weight, phototype, concurrent medications), cancer-related data (histology, topography), biopsy-related data (date, anesthesia modality, complications), treatment-related data (surgical procedure and related complications, chemotherapy regimen and related toxicity, radiotherapy characteristics: particle, energy, technique, fractionation, overall duration, target volume(s)). Comorbities that can affect IRS (e.g. diabetes, immunodeficiency, chronic inflammatory disease...) will be reported.

The following biological data from the radiobiological experiments will be reported in triplicates: γH2AX foci/nucleus prior irradiation and 24 h after, pATM foci foci/nucleus prior irradiation, 10 min and 1 h after, micronuclei/cell prior irradiation and 24 h after. 50 nuclei are counted for each condition.

Adverse events related to investigations (blood sample, skin biopsy or RT) as well as outcome will be recorded at RT simulation time, weekly during RT, at three months after RT, yearly during 5 years and every two years during the last 10 years (Table 1).

**Table 1:** Chronogram and investigations of the ARPEGE study

	Inclusion	Weekly follow-up during RT							3 months after RT	Once/year during the 5 first years	Every 2 years during the 10 next first		End of study (15 years)
Written Informed consent signature	X												
Checking of inclusion and non-inclusion criteria	X												
Inclusion	X												
Clinical exam	X	X	X	X	X	X	X	X	X	X	X	X	X
Medical history	X												
Biopsy	X												
Blood sample	X												
Toxicity recording		X	X	X	X	X	X	X	X	X	X	X	X
Biopsy-related adverse events recording		X	X	X	X	X	X	X	X	X	X	X	X
Blood sample-related adverse events recording		X	X	X	X	X	X	X	X	X	X	X	X
Concurrent treatments recording	X	X	X	X	X	X	X	X	X	X	X	X	X

### Anticipated completion of enrollment

All patients should be included over a 30-month period. The duration of follow-up is 3 months after the completion of the RT for the main objective and 15 years for the secondary objectives. The overall duration of the study is therefore 17.5 years.

Our current expectation is that the final patient will be enrolled by July 2019, and the entire study will be completed by July 2033. Cumulative enrollment reached 10 cases as of August 2017.

### Intervention

#### Biopsy

After information and written consent from the holder(s) of parental authority and/or the child/adolescent, a 2–5 mm skin biopsy will be sampled under general anesthesia (provided for another purpose, e.g. bone marrow biopsy, lumbar puncture, central venous catheter…) or local anesthesia once the indication of RT is certain. In populations B and C, whenever there is a definite indication of postoperative RT known at the time of surgery, the biopsy may be collected from the operative specimen or at the scar level without modifying the nature or extent of the procedure. In other cases, a 2 mm (12 G) punch biopsy will be sampled at the buttock. The biopsy aims to collect the epidermis and superficial dermis without ever coming into contact with the superficial muscular fascia. The use of iodized antiseptics is prohibited. The anonymized specimen will be sent fresh in 10 mL of appropriate culture medium at ambient temperature and within 48 h.

In parallel, 2.5 mL of venous blood may be collected to enrich a biological collection that will allow for further radiobiological studies (optional).

### Cell culture and irradiation

Patient-specific non-transformed fibroblast primo cultures will be raised. The cell lines will be irradiated (2 Gy) at early passage in the plateau phase of growth to mimic healthy tissues and to avoid any artifacts resulting from the cell cycle. The irradiated and control cells will be fixed at strategic times (10 min, 1 h, and 24 h).

### Immunofluorescence assay

Protocols for immunofluorescence with antibodies against pATM and γH2AX proteins and with DAPI counterstaining for scoring micronuclei have been described previously [15, 18]. The procedures will be repeated in triplicates and three independent experts from two centers will count the foci in blind to get a kinetic for each marker and classify each patient according to his/her IRS, which will remain hidden from the oncologist.

### Radiotherapy

RT will be planned according to state of the art recommendations without any individual adaptation. The dose delivered to the organs at risk will be systematically collected on the dose-volume histograms.

### Toxicity data collection

Early toxicity will be reported once a week during RT and then at 3 months, and rated on the NCI-CTACE v4.0 scale. Late toxicities, including second cancers, will be collected over 15 years and rated according to the same scale.

### Statistical analysis

#### Statistical methods

The discriminating ability of any IIF biomarker to predict the severity of toxicity will be defined by the area under the ROC (AUC) curve as well as its 95% confidence interval. The following hypothesis: H_0_: AUC ≤ 0.7 against H_1_: AUC > 0.7 will be tested.

The discriminating capacity of biomarkers will be compared by the nonparametric Mann-Whitney approach [19]. For each biomarker, the choice of the optimal threshold will be determined using the Youden index to maximize both sensitivity and specificity. The sensitivity and specificity of each biomarker will be compared by a Mac Nemar test. A multivariate logistic regression will be carried out on all biomarkers with a level of significance less than 0.2 in bivariate analyses. The simplification of this model will be carried out by a logistic multivariate regression with a downward selection at significance level 0.2 [20] using the bootstrap re-sampling method [21]. The discriminating capacity of the model will be estimated using the area under the ROC curve (95% confidence interval). The correlation between early and late toxicity will be evaluated with a Chi-Squared or Exact Fisher test.

The analysis of the main and secondary short-term objectives is planned as soon as all the included patients have 3 months of follow-up. Intermediate analysis of the long-term secondary objectives will be carried out at 2, 5 and 10 years.

### Power calculation

If we hypothesize an area under the ROC curve of 0.85 for the main endpoint, a 15% occurrence of early toxicity [22] and a risk of first specie at 2.5% then 222 patients are necessary to obtain a power greater than 0.8 including 10% of lost to follow-up. Thirty unusual early and late toxicities are therefore expected.

According to inter-regional data updated in 2015, the inclusion potential would be 150 patients per year in the territory.

## Discussion

From the basic research carried out by INSERM UMR1052 radiobiology group, Neolys Diagnostics proposes a powerful decision-making tool to the radiation oncology community in order to reduce side effects, while optimizing the treatment efficiency. With quasi optimal positive and negative predictive values, it is the only IRS test able to accurately quantify this trait according to a continuous spectrum with a strong biologic rationale when compared with other IRS assays. ARPEGE represents a unique opportunity to validate the skin IRS assay according to an appropriate methodology. To our knowledge, we lead the first study to document the specific distribution of IRS in the pediatric population.

The application on the pediatric population is relevant due to the scarcity of cancer prevalence and indications of RT, the specific tissue homeostasis in this population, and the major societal challenges of optimizing the quality of survival of children who will recover.

Due to the multiplicity of clinical situations in this heterogeneous population and in particular the protocols of concurrent chemotherapy we had considered to harvest the cells in the presence of drugs before irradiating them - in order to evaluate their radiosensitization potential. We abandoned this idea because of the over-cost, low feasibility and reproducibility, and bias on the constitutional trait pointed out by the assay.

With regard to the documents intended for children and their decision-making autonomy, it appeared necessary to cover all the differences in development. Using appropriate language and information materials, we always seek the agreement of the child. For this study, we developed 4 different information materials and consent forms for parents and children aged 13–17, 8–12 and under 8 years of age. A Childhood Cancer Parents Association validated the materials.

The duration of the study is compatible with an exhaustive collection of late toxicities, including radiation induced malignancies; The French expert centers have set up long-term monitoring structures in order to optimize the quality of survival. A stream wise recording of the dosimetric parameters performed routinely in France will provide new dose-volume-effect data for healthy tissues in pediatrics. Medico-economic data collected in an ancillary study on the same population will provide interesting information on the societal cost of sequelae induced by cancer treatments. Dose adaptation clinical trials integrating IRS a priori will be carried out in a second phase.

## Abbreviations

CTCAE: Common Terminology Criteria for Adverse Events
DSB: DNA double-strand breaks
IIF: Indirect immunofluorescence
IRS: Individual radiosensitivity
pATM: Phosphorylated isoform of ataxia telangiectasia mutated (ATM) protein
ROC: Receiver operating characteristic
RT: Radiotherapy
γH2AX: phosphorylated isoform of the histone variant H2AX
